# Endometrial Cancer Management in Young Women

**DOI:** 10.3390/cancers14081922

**Published:** 2022-04-11

**Authors:** Anna Markowska, Anita Chudecka-Głaz, Kazimierz Pityński, Włodzimierz Baranowski, Janina Markowska, Włodzimierz Sawicki

**Affiliations:** 1 Department of Perinatology and Womens’ Diseases, Poznan University of Medical Sciences, 60-535 Poznan, Poland; annamarkowska@vp.pl; 2 Department of Gynecological Surgery and Gynecological Oncology of Adults and Adolescents, Pomeranian Medical University, 70-204 Szczecin, Poland; 3 Department of Gynecology and Oncology, Jagiellonian University Medical College, 31-501 Krakow, Poland; pitynski@wp.pl; 4 Department of Gynecological Oncology, Military Institute of Medicine, 04-141 Warsaw, Poland; wbaranowski@yahoo.com; 5 The Clinic of Oncology, Poznan University of Medical Sciences, 60-569 Poznan, Poland; janina.markowska@oncology.am.poznan.pl; 6 Chair and Department of Obstetrics, Gynecology and Gynecological Oncology, Medical University of Warsaw, 02-091 Warsaw, Poland; saw55@wp.pl

**Keywords:** endometrial cancer, TCGA classification, ultrasound, MRI, hysteroscopy, progestogens, GnRH agonist

## Abstract

**Simple Summary:**

Endometrial cancer is a gynecological neoplasm characterized by a constant increase in incidence in highly developed countries. Due to the tendency towards late motherhood in these regions of the world, it is starting to affect more and more young women as well, often those who have not realized their maternity plans. At the same time, we note a very intensive development of research related to endometrial cancer, both in terms of diagnostics and new therapies. Therefore, nowadays, is there a sparing treatment option for young women?

**Abstract:**

Endometrial cancer (EC) rarely develops in young women. Most cases are associated with known risk factors: BMI > 30, history of Polycystic Ovary Syndrome (PCOs), and race differentiation. The molecular EC classification based on The Cancer Genome Atlas Research Network divides these heterogeneous cancers into four types: Polymerase Epsilon Mutation (POLE), Microsatellite Instability (MSI), Copy Number Low (CNL), and Copy Number High (CNH). This division was introduced to allow for early assessment of neoplastic changes and clinical management, including targeted therapies. The basic technique for imaging endometrium changes is transvaginal sonography. Hysteroscopy is the standard for obtaining endometrial material for histological evaluation. The MRI result permits assessment of the extent of EC cancer infiltration. In young women who want to preserve fertility, apart from surgery, conservative management is often implemented after strict selection based on clinical and pathological data. This pharmacological treatment involves the administration of progestogens MPA (medroxyprogesterone acetate) and MA (megestrol acetate). The use of metformin may increase the effectiveness of such treatment. An alternative option is to apply progestogens locally—via the levonorgestrel-releasing intrauterine device. In addition to pharmacological treatment, hysteroscopic resection may be used—part of the uterine muscle adjacent to the pathologically changed endometrium may also undergo resection. An alternative is the administration of estrogen receptor modulators (e.g., SERMs) or aromatase inhibitors, or GnRH agonists.

## 1. Introduction

According to global statistics, endometrial cancer (EC) is one of the most common cancers of the female genital organs; in terms of incidence, it ranks second after cervical cancer. In 2020, 417,367 women were diagnosed with EC worldwide, which accounts for 4.5% of all malignant neoplasms in women [1]. North America has the highest incidence, followed by Eastern Europe (21.1 and 20.2 per 100,000 women, respectively). The lowest incidence rate was found in Central Africa (2.3 per 100,000). EC deaths are more common in Eastern Europe (3.7/100,000) and lowest in Central Africa (0.8/100,000) [1].

Over 90% of endometrial cancer occurs in women above the age of 50, and the mean age of EC onset is 63 years [1,2]. 

EC in young women develops very rarely. Over the course of 12 years, the North American Society for Pediatric and Adolescent Gynecology identified several cases of EC in women under the age of 25 (3 cases among 69 diagnosed patients). A few cases of atypical endometrial hyperplasia were also found in this group of women (5 out of 69). Most cases of endometrial pathology in young women were associated with risk factors such as BMI > 30, smoking, and a history of PCOs (polycystic ovary syndrome) [3].

Another long-term study assessed the age of 551 EC patients. The exclusion criterion was the age of 60 [4]. In total, 18.7% were ≤40 years old in this group, and 81.3% of these women were between 41 and 60 years old. Age of ≤40 years was significantly associated with a higher BMI (*p* = 0.008). The incidence of synchronous ovarian cancer in young women was 9.2% vs. 0.7% in the 41–60 age group. 

Patient race seems to be correlated with the age at which EC develops [5]. A population analysis identified 35,850 women with EC aged <50 years. Among white women, there was a significantly lower percentage of patients <35 years old diagnosed with EC than among black women (7.8% vs. 13.6%) and a lower percentage of women aged 35–39 (13.6% vs. 15.6%). Moreover, in black women, EC was diagnosed at an advanced stage; the cancers were poorly differentiated, non-endometrioid. In multivariate models, the survival of these women was 19% worse than that of white women.

In 1983, Bokhman was the first to distinguish two types of EC, differing in etiology, age of onset, and clinical course and prognosis [6].

Type I—endometrioid EC is recognized in approximately 80% of patients and is associated with estrogen stimulation that is not balanced with progesterone. It has a slow course and a good prognosis. Type II—non-endometrioid EC, which includes serous, clear-cell, and undifferentiated carcinomas, is characterized by an aggressive course and poor prognosis. Type II EC affects an older age range of women in comparison to Type I.

These two types of EC are also associated with various genetic disorders [2,7,8,9,10]. In endometrioid endometrial carcinoma (EEC), the following are much more common:

1. Mutation in PTEN, a suppressor gene involved in the regulation of the cell cycle primarily via the PI3K/AKT signaling pathway, occurs in approximately 80% of ECs. PTEN mutations occur in Cowden syndrome, where the risk of developing EC is 5–10%.
2. Positive estrogen and progesterone receptors (ER, PR) in approximately 70% of cases,
3. a mutation in CTNNB1 in about 40%—which increases the mobility and invasiveness of cancer cells by reducing the expression of β-catenin.
4. Mutations in PIK3CA (phosphatidylinositol kinase) occur in about 50% of EC cases and affect the phosphorylation and activation of the AKT protein that inhibits apoptosis and promotes proliferation.
5. An increase in the frequency of microsatellite instability (approx. 20–45%), which is evidence of MMR dysfunction (mismatch repair) associated with a mutation in the MSH2, MLH1, MSH6, and PSM2 genes.

Mutations occur more frequently in EC type II: in the suppressor gene p53 (over 80%), p16 (around 45%), and overexpression of HER2 associated with cancer aggression (approximately 60%). Additionally, a decrease in E-cadherin function is more common, resulting in increased cell motility and invasion. In a meta-analysis based on electronic databases of 13,871 BRCA1/2 mutation carriers, there was a slightly increased risk of developing EC type II (RR 1.39; 95% CI 0.5–3.7) [11].

The lately developed diagnostic and prognostic possibilities, as well as targeted therapy, are based on molecular biology technique. According to these molecular markers, endometrioid carcinoma is characterized by frequent disruption of the PI3K-PTEN-AKT-mTOR, RAS-MEK-ERK signaling pathways, and the canonical WNT-β catenin pathway. Endometrial cancer shows the most mutations in the PI3K/AKT pathway among all neoplastic tumors. Single nucleotide variants in the HNF1B, KLF, EIF2AK, CYP19A1, SOX4, and MYC genes are strongly associated with this tumor and are involved in cell survival, estrogen metabolism, and transcription. The endocannabinoid system appears to contribute significantly to the growth and progression of endometrial cancer. Activation of the type 1 cannabinoid receptor inhibits the invasion of cancer cells, and endocannabinoids play an essential role in the inhibition of neoangiogenesis. Studies of circulating cell-free DNA (cfDNA) in women with endometrial cancer have shown that the relative length of cfDNA telomerase in women with endometrial cancer is shorter when compared to healthy women. These analyses suggest that the estimation of cfDNA may be a valuable tool for the early diagnosis of EC [12,13,14].

In 2013, the Cancer Genome Atlas Research Network (TCGA) research group, based on the analysis of 307 cases of endometrioid EC and 66 cases of serous and mixed cancers, using the method of microarrays and sequencing, presented a new molecular classification of endometrial cancer [15]. At the annual meeting of USCAP (the United States and Canadian Academy of Pathology), the value of this classification in the accuracy of risk prediction of endometrial cancer was confirmed [16].

## 2. Molecular Classification of Endometrial Cancer 

The molecular classification divides the EC into four subgroups: POLE, MSI, Copy Number Low (CNL), and Copy Number High (CNH).

POLE tumors with the POLE mutation (mutation in the DNA exonuclease domain of the epsilon-POL polymerase). These include endometrial carcinomas with ultra-mutation (frequency ≥ 100 mutations/Mb), a characteristic mutation signature (COSMIC signature 1), an increased immune response, and a very good prognosis, regardless of the clinical stage and histopathological type. In order to assess the significance of the identified variants, a scoring system was created, taking into account: C > A mutation above 20% (1 point), T > G mutation above 4% (1 point), indels below 5% (1 point), C > G mutation below 0.6% (1 point), TMB above 100 mut/Mb (1 point) and recurrent variants in endometrial cancer (1 point). Result >4 points means that it is a pathogenic POLE variant of the mutation; point score = 3 is a variant with unknown meaning; result <3 means a non-pathogenic POLE mutation. These tumors account only for 7% of cases.

MSI are tumors with high genetic instability, mainly low- and high-grade endometrioid types of carcinomas. They constitute 28% of the EC group. The MSI group contains tumors with ineffective mutator genes (MMR mismatch repair)—due to mutations, which makes it impossible to repair damaged DNA (MLH1, MSH2, MSH6, and PMS2). Mutations in PTEN and ARID1A and in the genes of the phosphatidylinositol (PIK3CA) family are less common [17,18,19,20,21]. This group includes cancers associated with Lynch Syndrome (3%). Women with Lynch Syndrome (LS) are younger compared to those with EC associated with sporadic mutations (47–55 years vs. 63 years) [22,23,24]. Identification of LS is essential because of the prevention offered to patients by close supervision from the age of 30 for people with MSH2 mutations and the age of 35 with MLH1 mutations. The use of checkpoint inhibitors is recommended in the treatment of these neoplasms [24,25,26,27].

Copy Number Low (CNL) are typical low-grade endometriotic neoplasms with low genomic copy number, low tumor mutation count (TMB), rare mutations in TP53, and frequent mutations in PTEN, PIK3CA, ARID1A, CTNNB1, and KRAS. They constitute 39% of all ECs.

Copy Number High (CNH) are mainly serous, and some high-grade endometriotic neoplasms have a high genomic copy number; frequent mutations in TP53 and PIK3CA, and rare in PTEN, have the worst prognosis. They account for 26% of EC [28,29].

Due to the difficulties in applying TCGA-based molecular classification in daily practice, the search for substitute classifications began. Initially, it seemed that the ProMisE (Proactive Molecular Risk Classifier for Endometrial Cancer) test, based on the detection of tumor DNA with ultrasensitive New Generation Sequencing (NGS), would be a prognostic and relatively simple test, facilitating the early assessment of neoplastic changes before the planned surgery. According to the authors publishing these data, this test was clinically promising [18]. However, the latest ESGO guidelines for the diagnosis and treatment of endometrial cancer provide a different classification, combining a surrogate of the primary molecular classification (surrogate biomarker assessment) with a known and used classification based on risk factors. The simplicity of this classification allows for its implementation in everyday use. Molecular classification surrogate—surrogate biomarker scoring is as follows:

MS—immunohistochemical assessment of MMRd.

Copy Number High—immunohistochemical evaluation of p53 mutations.

POLE—the necessity to use the NGS method to assess mutations, but with the permissible omission of this test in low-risk cancers.

Copy Number Low—NSMP, or nonspecific molecular profile, are endometrial carcinomas that cannot be classified into the remaining three subgroups.

The combination of surrogate biomarker assessment as a substitute for molecular classification with classification based on risk factors has been described in ESGO/ESTRO/ESP guidelines [24] considered high-grade. LVSI—lymph-vascular space invasion.

## 3. Imaging

### 3.1. USG (Ultrasonography) and MRI (Magnetic Resonance Imaging)

**Figure 1:**
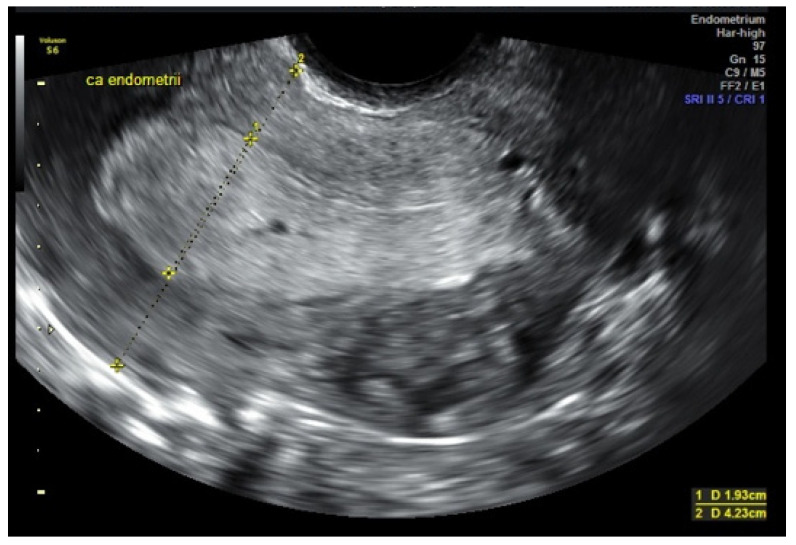
Transvaginal sonography (TVS imaging). Longitudinal section. Endometrial cancer, stage IA, G1, infiltration with homogeneous echogenicity.

Transvaginal ultrasonography is the primary imaging technique used to verify the uterine cavity in cases of abnormal bleeding and also enabling the assessment of the size of the neoplastic infiltration (tumor), the evaluation of myometrial invasion, and the cervical stroma [2,30] [Figure 1]. This technique has become a valuable tool in selecting patients for more or less radical surgical intervention or conservative fertility-sparing treatment.

It is believed that two methods currently used are comparable in terms of accuracy and efficacy—MRI and USG [31]. Studies published by Savelli et al. [32], Antonsen et al. [33], and Ortoft et al. [34] assessed the accuracy of both imaging methods in detecting myometrial and cervical infiltration. The accuracy value for MRI and USG was 66–82% and 72–84%, respectively, for myometrial, and 82–85% and 78–92%, respectively, for cervical stromal infiltration. These data also reflect the limitations of both methods, with a similar tendency to overestimate myometrial invasion and a tendency to underestimate cervical canal invasion. However, due to its wide availability, ultrasound remains the preferred method of imaging, while MRI should be used in more difficult cases, for example, with the coexistence of adenomyosis and fibroids.

A meta-analysis by Alcazár et al. [35] showed that the overall diagnostic accuracy of transvaginal ultrasound for the detection of deep myometrial invasion get a total sensitivity of 82% and specificity of 81%. Stromal infiltration of the cervical canal, as demonstrated by Mascilini et al. [36], is characterized by the loss of a clear border with the endometrium towards the cervical stroma, accompanied by increased perfusion of this region. It is estimated that the neoplastic infiltration assessment of the myometrium and cervix, both by ultrasound and by MRI, remains inadequate in 15–25% of cases [30,36].

### 3.2. Ultrasound Prognosis of the Histological Type

As the histological malignancy of cancer (G2, G3) increases, its tendency to infiltrate deeper layers of the uterine muscle also becomes greater. In low-grade tumors, infiltration of the uterine muscle was found in less than 20% of cases, while in the high-grade tumors in over 50%. Thus, higher clinical advancement correlates with a more pronounced negative prognostic significance of low cancer maturity [30].

**Figure 2:**
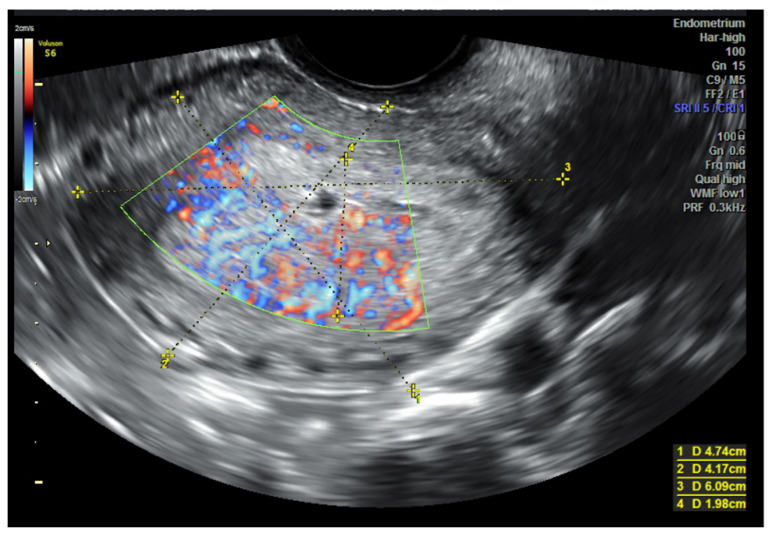
Transvaginal sonography (TVS imaging). Longitudinal view. IB, G3 endometrial cancer. Variable echogenicity of the infiltration, with remarkably intensified vascularization—4 points according to IETA.

The discrepancy between the preoperative and final histopathological examination in endometrial cancer is estimated to be at up to 40% of cases. The main reason for that may be the heterogeneity of neoplastic tissues and often insufficient representativeness of samples obtained during the initial diagnostic procedure [37]. This is of particular importance in cases qualified for fertility-preserving procedures. A multicenter prospective study conducted by Epstein et al. [38] in 2007–2009 identified sono-morphological and Doppler features characteristic of low-grade endometrial cancer and also cases with a high risk of recurrence. These results were confirmed by Fischerov et al. in another prospective study [39]. They showed that low-risk endometrial cancers (G1, IA) most often showed homogeneous echogenicity, with no or minimal intensity of vascularization (1–2 points according to IETA), while tumors of heterogenous echogenicity showed profuse perfusion (3–4 points according to IETA) [Figure 2]. On the other hand, tumors of heterogenous echogenicity with profuse perfusion (3–4 points according to IETA) and numerous multifocal vessels penetrating from the myometrial tissue into the infiltration area more often characterized low-differentiated (high-grade) cancers (G2, G3) and those showing deep myometrial and/or the stroma of the cervix infiltration. Therefore, as the authors emphasize, these parameters can be additionally used in the qualification for more radical surgery, and in the case of young women, it would be helpful to consider conservative treatment options. Based on the above studies, the degree of vascularization extent can be considered a valuable additional marker determining the biology of this neoplasm. As the depth of infiltration increases and the histological maturity decreases, the color Doppler signal is recorded statistically significantly more often. This is related to the described in many papers observations that the greater number of lymph node metastases is correlated with histological malignancy markers and with the depth of uterine invasion. Therefore, it can be assumed that the intensity of angiogenesis may be expressed by the biological grade of malignancy. The direct relationship between the intensity of angiogenesis and high histological grade suggests that it could be an additional prognostic factor [40].

## 4. Invasive Diagnostic Procedures

Although modern and advanced TVS enables detailed imaging of uterine structures, this technique does not provide samples for pathomorphological diagnosis. Hysteroscopy is a minimally invasive procedure that allows direct visual evaluation of the cervical canal and uterine cavity and also allows the collection of material for pathomorphological evaluation. Hysteroscopy is considered a simple and safe method and is therefore considered the primary technique among minimally invasive procedures. Contemporary hysteroscopy is used in many clinical indications [41].

In the group of women under 40, endometrial cancer can be unexpectedly detected during the hysteroscopic examination due to unsuccessful embryo transfer procedures. In women with a history of unsuccessful assisted reproductive procedures, hysteroscopy often detects endometrial polyps, submucosal fibroids, adhesions, and uterine congenital abnormalities (e.g., septum). Contrary to these pathologies, the percentage of malignant lesions of the endometrium and their impact on fertility are not known due to the lack of reports based on a large group of the patients [42]. Hysteroscopy is widely used to detect and remove polyps, with a sensitivity of 93% and a specificity of 90%. The abundant vascularization, the presence of ulcers, and the irregular surface of the polyp during hysteroscopic visualization indicate the malignant nature of the lesion (with a sensitivity of 96% and a specificity of 93.5%) [43]. Endometrial cancer diagnosed in women under 40 years of age is usually a highly differentiated focal endometrioid tumor confined to the endometrium or with minimal myometrial invasion, with high expression of estrogen and progesterone receptors. Clinical observations show that 8% of patients under 40 with endometrial cancer are diagnosed with stage I (50–90%). The most commonly identified risk factors in this group of women are obesity, usually after the age of 20, physical inactivity, higher fasting insulin levels, type II diabetes, hypertension, early menarche, Lynch Syndrome, and anovulatory cycles occurring, e.g., in polycystic ovary syndrome [44].

The appearance of small-diameter (4–5 mm) hysteroscopes allows outpatient imaging without general anesthesia. Apart from the possibility of relocation of neoplastic cells to the peritoneal cavity, diagnostic hysteroscopy carries a small risk of complications [41,45,46].

In a multicenter study conducted by Garuti et al. [47], hysteroscopy in the diagnosis of endometrial cancer was characterized by a sensitivity of 54.2%, specificity of 47.2%, and accuracy of 54%. A study by the same author published in 2001 indicated the high value of hysteroscopy in the differentiation of normal and abnormal endometrial histopathology. The sensitivity of hysteroscopic sampling was 94.2%, with a specificity of 88.8%, and negative and positive predictive value was 96.3% and 83.1%, respectively. Previous systematic reviews of published studies showed an overall sensitivity and specificity of hysteroscopic sampling for endometrial cancer detection of 86.4% and 99.2%, respectively. In cases of atypical endometrial hyperplasia, which is generally believed to precede the onset of endometrial carcinoma, the sensitivity, positive predictive value, and negative predictive value were calculated at 90.4%, 58.4%, and 86.6%, respectively [48]. Narrowband imaging possibilities of hysteroscopes significantly increase the sensitivity of the examination to 97.2%, with a specificity similar to conventional hysteroscopy [49]. The subjectivity of hysteroscopic imaging, depending on the experience of the examiner, does not allow for proper differentiation of endometrial changes. Cancer that is polypoid in appearance can be mistaken for a benign endometrial polyp. The visual diagnosis is based primarily on the presence of visible abnormalities of the uterine cavity caused by the presence of focal or extensive nodular, polypoid, papillary, and mixed lesions, as well as focal necrosis, the fragility of the endometrium, and atypical vessels. Therefore, four types of hysteroscopic pictures of endometrial cancer have been proposed, i.e., polypoid, nodular, papillary, and ulcerative [50].

To objectify the hysteroscopic examination and improve detection, it was proposed to use the endometrial cancer point scale. However, despite the described greater accuracy, the introduction of the scoring system requires confirmation of its usefulness for a large group of patients and in various clinical situations [50].

After the introduction of hysteroscopy in the endometrial cancer diagnosis, the problem of the oncological safety of this technique was raised. Hysteroscopy can cause cancer cells to enter the peritoneal cavity through the Fallopian tubes. It is especially important in women of childbearing age who usually have patent Fallopian tubes. Endometrial cancer cell migration increases the risk of iatrogenic tumor spreading. However, cancer cells may be functionally able to adhere to a matrix within the peritoneal cavity, but reviews and meta-analyses of the studies published so far showed the safety of hysteroscopy in the diagnosis of endometrial cancer and no significant impact on the prognosis in the early stage of this cancer was shown [51].

## 5. Fertility Sparing

### 5.1. Eligibility for Conservative Treatment

Fertility-preserving treatment is possible in a selected group of young women, but only with their approval and after a thorough explanation of the risks associated with it. If the patient does not consent to such treatment, we should use standard methods of treatment.

Although surgery is the treatment of choice in women with atypical endometrial hyperplasia and endometrial cancer, in women wishing to maintain fertility, alternative treatment with uterine preservation may be considered, but certain conditions must be met—endometrioid endometrial cancer stage FIGO IA G1/G2 [52,53]. In the selection of patients eligible for the conservative treatment of AEH and EC, molecular classification has been recently proposed in addition to a routine histopathological assessment of preparations [24,54]

Based on the TCGA, two systems for the practical clinical application of the assessment of molecular changes in the therapeutic management of patients with endometrial cancer have been developed (see the previous section).

Previous studies have shown that these assessments were predictive and correlate both with the effectiveness of pharmacological treatment of AEH and EC in patients who wish to maintain fertility as well as with the risk of disease recurrence. Therefore, the molecular status assessed in the Proactive Molecular Risk Classifier for Endometrial Cancer system significantly affects the effectiveness of conservative treatment and may be a qualifying factor for this type of treatment [55,56].

### 5.2. Treatment with Progestogens

17-alpha progesterone derivatives, i.e., medroxyprogesterone acetate (MPA) and megestrol acetate (MA), are the first-line drugs in the fertility-preserving treatment of atypical endometrial hyperplasia and early stage of endometrial cancer; hydroxyprogesterone caproate is used rarely (less than 1%). The use of other progestogens is not very popular—it accounts for approximately 13.5% of all patients and is of rather historical importance [53].

MPA doses range from 20 to 1500 mg/day, and 400–600 mg/day is most often cited in the literature. Megestrol acetate is used in doses ranging from 40 to 480 mg/day—most often 160–320 mg/day [57]. The treatment regimen used is continuous progestogen administration, although a 14-day cycle during the second phase of the menstrual cycle has also been reported. The recommended duration of treatment with progestogens is an average of 6 months, although the latest clinical data indicate that it is reasonable to use progestogens for up to 12 months—during this period, the therapeutic effect (complete remission—CR) occurs in 80–90% of patients. The treatment monitoring requires a histopathological assessment of the endometrium at intervals of 2–3 months. Various techniques of obtaining endometrial samples are used—aspiration biopsy, curettage of the uterine cavity, and hysteroscopic targeted endometrial sampling [57].

An additional method to increase the effectiveness of progestogen therapy is metformin administration. The use of this drug is based on epidemiological data that indicate significantly better oncological outcomes (overall survival—OS, recurrence rate—RR) in patients after endometrial cancer treatment, especially in the group of patients with accompanying diabetes. The effectiveness of metformin is due to its inhibition of MAP (mitogen-activated protein kinase—MAPK), blocking the activity of cyclin D1, reducing the expression of H19 non-coding RNA, increasing the silencing of genes (including H19), and decreasing the expression of mRNA encoding PPP2R4. Another clinically beneficial effect of metformin is the reduction of insulin resistance, the improvement of the quality of ovulation, and the inhibition of body weight gain caused by the administration of high doses of MPA. This form of therapy is especially recommended in obese patients [58].

According to the results of clinical trials, metformin is administered in a dose from 750 mg/day to 2250 mg/day. In 2019, a prospective randomized clinical trial under the acronym FELICIA was registered in Japan. In this ongoing study, patients aged between 20 and 42 with atypical endometrial hyperplasia or cancer (FIGO IA) receive MPA at a daily dose of 600 mg for 32 weeks. Additionally, patients can take 100 mg/day of acetylsalicylic acid as prophylaxis against thromboembolism. The patients have been divided into the MPA only group (control)—40 patients, the MPA and metformin group at the initial dose of 500 mg and which after a month is increased to 750 mg—40 patients, and the MPA and metformin group at the initial dose of 500 mg, increased monthly to 1500 mg/day—40 patients. Treatment effects are monitored based on the histopathological assessment of the endometrium obtained from abrasion performed every eight weeks. After the end of MPA therapy, the administration of metformin is continued until the conception or recurrence of the disease. Histopathological assessment—endometrial sampling every three months (aspiration, hysteroscopy) is performed for the next three years. The planned study period is 5.5 years. The results of this study will help to optimize the simultaneous use of MPA and metformin in terms of oncological and reproductive outcomes [59].

### 5.3. Levonorgestrel Intrauterine System (LNG-IUS)

High doses of oral progestogens are associated with a significant risk of side effects (weight gain, edema, hypertension, depressed mood, gastric disorders, and coagulation disorders). In order to avoid the systemic effects of progestogens administered in such high doses, an alternative method of treatment is to use a levonorgestrel intrauterine system (LNG IUS) that releases levonorgestrel—a second-generation progestogen derivative of 19-nortestosterone. The therapeutic system releases locally high doses (up to 20 µg/d) of a strong progestogen, causing decidualization and endometrial atrophy. This type of treatment, unlike oral therapy, is well-tolerated. There are also data on combination therapy with LNG IUS and oral administration of MPA 500 mg/d. The recommended treatment period is at least 9 months (57). Another type of combined therapy is the use of LNG IUS and GnRH analogs [53]. In the feMMe study, which included a specific group of patients with AEH and EC with a BMI >40 kg/m^2^, treatment was applied using LNG IUS with metformin and weight loss recommendation [60].

### 5.4. The Role of Hysteroscopy in the Diagnosis and Treatment of AEH and EC

In addition to pharmacological treatment, hysteroscopic endometrial resection is a recommended, albeit not independent, treatment method for atypical endometrial hyperplasia and endometrial cancer FIGO IA G1/2. The technique of hysteroscopic excision of endometrial lesions was first described in 2010. In a prospective study involving a small group (six patients), the three-stage excision method was used to remove uterine lesions. The first step was the removal of the lesion itself, and the second step was to remove the endometrium adjacent to the previously removed pathological focus. Finally, the third stage was to excise the myometrium fragment directly under the endometrial lesion. Patients after hysteroscopic resection received 160 mg/d of MA for 12 months. During monitoring, diagnostic hysteroscopy performed every 3 months did not reveal any endometrial pathology in any patients. Furthermore, from a group of six patients, four (66%) gave birth to healthy children [61]. In another study, after the three-stage hysteroscopic resection of the lesion in 14 patients, eight patients were treated with MA at a dose of 160 mg/day for 6 months, and six patients received LNG IUS for 1 year. During the follow-up (13–79 months, average 40 months), only one patient had a recurrence of the disease 5 months after hysteroscopic resection and underwent a hysterectomy. The longest observations of patients after hysteroscopic EC resection and MA treatment are 16 years. In this group of patients, no recurrence of the disease was found. The largest data on the primary hysteroscopic treatment of AEH (120 patients) and EC (40 patients) and then treated with progestogens were published in a retrospective study by Yang et al. [62]. Only in three patients with AEH and in one of the EC patients the disease progressed. Complete remission of lesions was found after 6.7 (1–18) months of treatment, and out of 60 wishing to become pregnant, 23 (38.3%) achieved reproductive success. In a study by Giampaolino et al. [63], it was shown that hysteroscopic resection of pathological changes in the uterus followed by treatment with progestogens (systemic, intrauterine) produces similar reproductive results but significantly reduces the risk of tumor recurrence.

Another treatment method for AEH and EC is the use of drugs that affect estrogen by blocking its receptors (SERM, SERD) or lowering the concentration of endogenous estrogens (aromatase inhibitors, GnRH agonists). The few data currently published are very promising, but this method requires further research. In a pilot study with GnRH agonists and aromatase inhibitors in six women with BMI >30 kg/m^2^, complete remission of the disease was found in all patients within 3–6 months. In total, 75% of patients became pregnant, and 50% gave live birth [64].

In addition to pharmacological treatment, it has been proposed that young women with EC receive psychological support as they are at high risk of developing anxiety and depression, which have a negative influence on the quality of life [65].

## 6. Obstetric Outcomes

Fertility-sparing treatment of EC and AEH is feasible, and selected women can satisfy their reproductive wishes. This kind of treatment should not be contraindicated in older patients with previous infertility or obesity [66,67]. A meta-analysis published in 2012 was based on thirty-four observational studies, evaluating the regression, relapse, and live birth rates of early-stage EC (408 women) and ACH (151 women) with fertility-sparing treatment. Fertility-sparing treatment for EC achieved a pooled regression rate of 76.2%, a relapse rate of 40.6%, and a live birth rate of 28% [66]. Another analysis based on 24 studies and 370 patients show the 12- and 24-month remission rate were 78.0% and 81.4%, respectively. The 12- and 24-month recurrence probabilities were 9.6% and 29.2%, respectively. In total, 22 studies totaling 351 patients were used to assess the pregnancy rate; 111 subjects (32%) had one pregnancy or more [67].

## 7. Conclusions

In light of the published data available, new trials based on molecular classification may lead to progress in fertility-sparing conservative treatment. Different molecular groups have various levels of prognosis and could be a factor in selecting treatment for individual patients, especially young women, alongside standard procedures already present in common practice.
